# Supplementary material for: The Abnormal Accumulation of Lipopolysaccharide Secreted by Enriched Gram-Negative Bacteria Increases the Risk of Rotavirus Colonization in Young Adults
Source: Microorganisms. 2023 Sep 11;11(9):2280. doi: 10.3390/microorganisms11092280 (PMC10535061; doi:10.3390/microorganisms11092280)
Supplement: Supplementary file 1 [file microorganisms-11-02280-s001.zip › File S1. Instruction for ELISA kit.pdf]

本试剂盒只能用于科学研究，不得用于医学诊断

人轮状病毒（RV）ELISA 检测试剂盒

使用说明书

检测原理

试剂盒采用双抗体夹心法酶联免疫吸附试验（ELISA）。往预先包被人轮状病毒（RV）捕获抗体的包被微孔中，依次加入阴性对照、阳性对照、样本、HRP标记的检测抗体，经过温育并彻底洗涤。用底物TMB显色，TMB在过氧化物酶的催化下转化成蓝色，并在酸的作用下转化成最终的黄色。颜色的深浅和样品中的人轮状病毒（RV）呈正相关。用酶标仪在450nm 波长下测定吸光度（OD 值），判断样品是否含有人轮状病毒（RV）。

样品收集、处理及保存方法

1. 血清：使用不含热原和内毒素的试管，操作过程中避免任何细胞刺激，收集血液后，3000 转离心 10 分钟将血清和红细胞迅速小心地分离。
2. 血浆：EDTA、柠檬酸盐或肝素抗凝。3000 转离心 30 分钟取上清。
3. 细胞上清液：3000 转离心 10 分钟去除颗粒和聚合物。
4. 组织匀浆：将组织加入适量生理盐水捣碎。3000 转离心 10 分钟取上清。
5. 保存：如果样本收集后不及时检测，请按一次用量分装，冻存于-20℃，避免反复冻融，在室温下解冻并确保样品均匀地充分解冻。

自备物品

1. 酶标仪（450nm）
2. 高精度加样器及枪头：0.5-10uL、2-20uL、20-200uL、200-1000uL
3. 37℃恒温箱

操作注意事项

1. 试剂盒保存在 2-8℃，使用前室温平衡 20 分钟。从冰箱取出的浓缩洗涤液会有结晶，这属于正常现象，水浴加热使结晶完全溶解后再使用。
2. 实验中不用的板条应立即放回自封袋中，密封（低温干燥）保存。
3. 预处理后的样本无需稀释，直接取 10μL 加样即可。
4. 严格按照说明书中标明的时间、加液量及顺序进行温育操作。
5. 所有液体组分使用前充分摇匀。

试剂盒组成

名称	96 孔配置	48 孔配置	备注
微孔酶标板	96 孔	48 孔	无
阴性对照	0.3mL	0.3mL	无
阳性对照	0.3mL	0.3mL	无
样本稀释液	6mL	3mL	无
检测抗体-HRP	10mL	5mL	无

20×洗涤缓冲液	25mL	15mL	按说明书进行稀释
底物 A	6mL	3mL	无
底物 B	6mL	3mL	无
终止液	6mL	3mL	无
封板膜	2 张	2 张	无
说明书	1 份	1 份	无
自封袋	1 个	1 个	无

试剂的准备

20×洗涤缓冲液的稀释：蒸馏水按 1：20 稀释，即 1 份的 20×洗涤缓冲液加 19 份的蒸馏水。

洗板方法

1. 手工洗板：甩尽孔内液体，每孔加满洗涤液，静置 1min 后甩尽孔内液体，在吸水纸上拍干，如此洗板 5 次。
2. 自动洗板机：每孔注入洗液 350μL，浸泡 1min，洗板 5 次。

操作步骤

1. 从室温平衡 20min 后的铝箔袋中取出所需板条，剩余板条用自封袋密封放回 4℃。
2. 设置阴、阳性对照孔和样本孔，阴、阳性对照孔中加入阴性对照、阳性对照各 50μL；
3. 待测样本孔先加待测样本 10μL，再加样本稀释液 40μL；
4. 随后阴、阳性对照孔和样本孔中每孔加入辣根过氧化物酶（HRP）标记的检测抗体 100μL，用封板膜封住反应孔，37℃水浴锅或恒温箱温育 60min。
5. 弃去液体，吸水纸上拍干，每孔加满洗涤液，静置 1min，甩去洗涤液，吸水纸上拍干，如此重复洗板 5 次（也可用洗板机洗板）。
6. 每孔加入底物 A、B 各 50μL，37℃避光孵育 15min。
7. 每孔加入终止液 50μL，15min 内，在 450nm 波长处测定各孔的 OD 值。

结果判断

1. 试验有效性：阳性对照孔 OD 值平均值≥1.00；

阴性对照孔 OD 值平均值≤0.15。

2. 临界值（Cut off）计算：临界值=阴性对照孔平均值+0.15

3. 阴性判断：样品 OD 值＜临界值（Cut off），样品为阴性

4. 阳性判断：样品 OD 值＞临界值（Cut off），样品为阳性

试剂盒性能

1. 准确性：阳性对照孔 OD 值平均值≥1.00；阴性对照孔 OD 值平均值≤0.15，说明试验结果有效。
2. 特异性：不与其它可溶性结构类似物交叉反应。
3. 重复性：板内、板间变异系数均小于 15%。
4. 贮藏：2-8℃，避光防潮保存。
5. 有效期：6 个月

免责声明

1. 试剂盒仅供研究使用，不得用于临床实验或人体实验，否则所产生的一切后果，由实验者承担，本公司概不负责。

2. 严格按照说明书操作，实验者违反说明书操作，后果由实验者承担。

FOR RESEARCH USE ONLY.
NOT FOR USE IN DIAGNOSTIC PROCEDURES.

Human rotavirus (RV) ELISA Kit instruction

Intended use

This RV ELISA kit is intended Laboratory for Research use only and is not for use in diagnostic or therapeutic procedures. In order to determine whether contains RV in the sample, this RV ELISA Kit includes Negative control and Positive control. The Stop Solution changes the color from blue to yellow and the intensity of the color is measured at 450 nm using a spectrophotometer. The color depth was positively correlated with the RV in the sample .

Sample collection and storages

Serum - Use a serum separator tube and allow samples to clot for 30 minutes before centrifugation for 10 minutes at approximately 3000×g. Remove serum and assay immediately or aliquot and store samples at -20℃ or -80℃.Avoid repeated freeze-thaw cycles

Plasma - Collect plasma using EDTA or heparin as an anticoagulant. Centrifuge samples for 30 minutes at 3000×g at 2-8℃ within 30 minutes of collection. Store samples at -20℃or -80℃. Avoid repeated freeze-thaw cycles.

Cell culture supernates and other biological fluids - Remove particulates by centrifugation and assay immediately or aliquot and store samples at -20℃or -80℃. Avoid repeated freeze-thaw cycles.

⚠ **Note:** The samples should be centrifugated adequately and no hemolysis or granule was allowed.

Materials required but not supplied

1. Standard microplate reader(450nm)
2. Precision pipettes and Disposable pipette tips.
3. 37 ℃ incubator

Precautions

1. Do not substitute reagents from one kit to another. Standard, conjugate and microplates are matched for optimal performance. Use only the reagents supplied by manufacturer.
2. Do not remove microplate from the storage bag until needed. Unused strips should be stored at 2-8°C in their pouch with the desiccant provided.
3. Mix all reagents before using.

Remove all kit reagents from refrigerator and allow them to reach room temperature (20-25°C)

Materials supplied

Name	96 determinations	48 determinations
Microelisa stripplate	96 strips	48 strips
Negative control	0.3ml	0.3ml
Positive control	0.3ml	0.3ml
Sample diluent	6.0ml	3.0ml
HRP-Conjugate reagent	10.0ml	5.0ml
20X Wash solution	25ml	15ml
Chromogen Solution A	6.0ml	3.0ml
Chromogen Solution B	6.0ml	3.0ml
Stop Solution	6.0ml	3.0ml
Closure plate membrane	2	2
User manual	1	1
Sealed bags	1	1

Reagent preparation

20×wash solution:Dilute with Distilled or deionized water 1:20.

Assay procedure

1. Prepare all reagents before starting assay procedure. It is recommended that all Standards and Samples be added in duplicate to the Microelisa Stripplate.

2. Separately add Positive control and Negative control 50µl to the Positive and Negative well, Add testing sample 10µl Then add sample diluent 40µl to testing sample well; Blank well doesn't add anyting.

3. Add 100µl of HRP-conjugate reagent to each well, cover with an adhesive strip and incubate for 60 minutes at 37°C.

4. Aspirate each well and wash, repeating the process four times for a total of five washes. Wash by filling each well with Wash Solution (400µl) using a squirt bottle, manifold dispenser or autowasher. Complete removal of liquid at each step is essential to good performance. After the last wash, remove any remaining Wash Solution by aspirating or decanting. Invert the plate and blot it against clean paper towels.

5. Add chromogen solution A 50µl and chromogen solution B 50µl to each well. Gently mix and incubate for 15 minutes at 37°C. **Protect from light.**

6. Add 50µl Stop Solution to each well. The color in the wells should change from blue to yellow. If the color in the wells is green or the color change does not appear uniform, gently tap the plate to ensure thorough mixing.

7. Read the Optical Density (O.D.) at 450 nm using a microtiter plate reader within 15 minutes.

Determine the result

1. Test validity: the average of Positive control well≥1.00; the average of Negative control well ≤0.15.

2. Calculate Critical (CUT OFF): Critical= the average of Negative control well + 0.15.

Negative Result: sample OD< Calculate Critical (CUT OFF) is Negative.

Positive Result: sample OD≥ Calculate Critical (CUT OFF) is Positive.

Storage and validity

Storage： 2-8℃.
validity： six months.

FOR RESEARCH USE ONLY;
NOT FOR THERAPEUTIC OR DIAGNOSTIC APPLICATIONS!
PLEASE READ THROUGH ENTIRE PROCEDURE BEFORE BEGINNING!
