# Supplementary material for: The Abnormal Accumulation of Lipopolysaccharide Secreted by Enriched Gram-Negative Bacteria Increases the Risk of Rotavirus Colonization in Young Adults
Source: Microorganisms. 2023 Sep 11;11(9):2280. doi: 10.3390/microorganisms11092280 (PMC10535061; doi:10.3390/microorganisms11092280)
Supplement: Supplementary file 1 [file microorganisms-11-02280-s001.zip › File S2. qPCR protocol for HRV detection.pdf]

Protocol of real-time PCR to detect HRV

RNA was extract from stool sample using Mag-MK Virus RNA Extraction Kit (Sangon, Shanghai, China), followed by using MightyScript First Strand cDNA Synthesis Master Mix (Sangon, Shanghai, China) to synthesize the first-strand cDNA as template. The volume of qPCR reaction was 30 μL, with the following ingredients: 0.3 μL of each primer (10 μM), 0.2 μL of TaqMan probe (10 μM), 1 μL of template, 10 μL of TaqProbe qPCR-Multiplex Mastermix (Sangon, Shanghai, China), and the remaining volume with nuclease-free water. The amplification on Roche LightCycler 96 Instrument (Roche Life Science, Basel, Switzerland), with the following program: 95 ◦C for 10 m, following by 45 cycles of 95 ◦C for 10 s, 59 ◦C for 10 s, and 72 ◦C for 20 s. Fluorescence signals were detected at the end of each cycle of the extension step.

The sequence of the primers

Forward primer: 5'-ACRTRACCCTCTATGAGCACA-3'

Reverse primer: 5'-GGTCACATAACGCCCCTATA-3'

Probe: ROX-AGTTAAAAGCTAACACTGTCAAAAACCTAA-BHQ2*
