# Supplementary material for: The Abnormal Accumulation of Lipopolysaccharide Secreted by Enriched Gram-Negative Bacteria Increases the Risk of Rotavirus Colonization in Young Adults
Source: Microorganisms. 2023 Sep 11;11(9):2280. doi: 10.3390/microorganisms11092280 (PMC10535061; doi:10.3390/microorganisms11092280)
Supplement: Supplementary file 1 [file microorganisms-11-02280-s001.zip › File S3. The simplified food questionnaire.pdf]

The simplified food-frequency questionnaire

1. Basic information:

   Name________ Age______ weight_______

2. Dietary habit (including food categories and items): __________

   Your favorite foods (multiple selection): a: none; b: Meat (pork, beef, mutton and poultry); c: fish (fish, shrimp, crab); d: fresh vegetables, e: legumes and products; f: rice and wheat flour; g: fruits; h: eggs; I: milk and products.

3. Intake of rich and chow: a: every day; b: 5~6 times a week; c: 3~4 times a week; d: 1~2 times a week; e: never; How many bowls to eat: _______bowls

4. Intake of wheat flour (including steamed bun, steamed stuffed bun, noodle, dumplings, bread): a: every day; b: 5~6 times a week; c: 3~4 times a week; d: 1~2 times a week; e: never;

5. Intake of milk and products (including milk, yogurt, powdered milk): a: every day; b: 5~6 times a week; c: 3~4 times a week; d: 1~2 times a week; e: never; How many bottles to drink: _______bottles

6. Intake of fish (including fish, shrimp, crab): a: every day; b: 5~6 times a week; c: 3~4 times a week; d: 1~2 times a week; e: never; How much to eat:______

7. Intake of eggs: a: every day; b: 5~6 times a week; c: 3~4 times a week; d: 1~2 times a week; e: never; How much to eat:______

8. Intake of meat (pork, beef, mutton and poultry): a: every day; b: 5~6 times a week; c: 3~4 times a week; d: 1~2 times a week; e: never; How much to eat:________

9. Intake of fresh vegetables: a: every day; b: 5~6 times a week; c: 3~4 times a week; d: 1~2 times a week; e: never; How much to eat:______

10. Intake of fruits: a: every day; b: 5~6 times a week; c: 3~4 times a week; d: 1~2 times a week; e: never; How much to eat:______

11. Legumes and products: a: every day; b: 5~6 times a week; c: 3~4 times a week; d: 1~2 times a week; d never; How much to eat: ________
