# Supplementary material for: The Abnormal Accumulation of Lipopolysaccharide Secreted by Enriched Gram-Negative Bacteria Increases the Risk of Rotavirus Colonization in Young Adults
Source: Microorganisms. 2023 Sep 11;11(9):2280. doi: 10.3390/microorganisms11092280 (PMC10535061; doi:10.3390/microorganisms11092280)
Supplement: Supplementary file 1 [file microorganisms-11-02280-s001.zip › Table S1.pdf]

**Table S1.** Baseline characteristics of Participants

| | Gender % (Prevalence) | Age (year) (mean ± SD) | BMI kg/cm$^2$ (mean ± SD) | Positive rate (%) (Prevalence) |
|---|---|---|---|---|
| Male | 53.3% (160/300) | 20.6 ± 0.8 | 20.2 ± 2.4 | 13.8% (22/160) |
| Female | 46.7% (140/300) | 21.2 ± 0.5 | 22.3 ± 3.2 | 10.7% (15/140) |
| Total | 100% (60/60) | 21.6 ± 0.8 | 21.4 ± 2.7 | 12.3% (37/300) |
