# Supplementary material for: The Abnormal Accumulation of Lipopolysaccharide Secreted by Enriched Gram-Negative Bacteria Increases the Risk of Rotavirus Colonization in Young Adults
Source: Microorganisms. 2023 Sep 11;11(9):2280. doi: 10.3390/microorganisms11092280 (PMC10535061; doi:10.3390/microorganisms11092280)
Supplement: Supplementary file 1 [file microorganisms-11-02280-s001.zip › Table S2.pdf]

Table S2: Adjusted ORs (95% CI) for HRV colonization according to quartiles of exposure for urine antibiotics in single-antibiotic model

		Quartile 1	Quartile 2	Quartile 3	Quartile 4	p trend
PVAs	**Tetracycline hydrochloride**					
	Range	< 0.39	0.39~0.45	0.46~0.64	≥0.65	
	Mode 1	1.00 (ref)	1.00(0.00~∞)	3.23E(0.00~∞)	2.61E(0.00~∞)	0.022
	**Doxycycline hydrochloride**					
	Range	< 0.32	0.33~0.56	0.57~0.62	≥0.63	
	Mode 1	1.00 (ref)	0.00(0.00~∞)	0.00(0.00~∞)	0.00(0.00~∞)	0.022
	**Oxytetracycline hydrochloride**					
	Range	< 0.92	0.92~1.00	1.01~1.08	≥1.09	
	Mode 1	1.00 (ref)	2.00 （0.05~78.25）	0.00(0.00~∞)	0.50 （0.01~19.56）	0.384
	**Sulfametoxydiazine**					
	Range	< 1.2	1.30~1.51	1.52~1.68	≥1.69	
	Mode 1	1.00 (ref)	0.00(0.00~∞)	0.00(0.00~∞)	0.00(0.00~∞)	
	**Norfloxacin**					
	Range	< 0.74	0.75~0.83	0.83~0.90	≥0.91	
	Mode 1	1.00 (ref)	0.00(0.00~∞)	0.00(0.00~∞)	0.00(0.00~∞)	0.103
	**Sulfamethoxazole**					
	Range	< 1.032	1.03~1.16	1.17~1.30	≥1.31	
	Mode 1	1.00 (ref)	0.00(0.00~∞)	0.00(0.00~∞)	0.00(0.00~∞)	0.022
	**Ofloxacin**					
	Range	< 0.91	0.91~1.00	1.01~1.08	≥1.09	
	Mode 1	1.00 (ref)	2.00 （0.05~78.25）	0.00(0.00~∞)	0.50 （0.01~19.56）	0.384
	**Penicillin V**					
	Range	< 0.00	0.00~0.00	0.00~5.01	≥5.02	
	Mode 1	1.00 (ref)	0.41 (0.35~0.81)	0.73 (0.52~1.32)	0.60 (0.45~1.07)	0.283
	**Lomefloxacin hydrochloride**					
	Range	< 0.91	0.00~0.00	0.00~8.15	≥8.16	
	Mode 1	1.00 (ref)	1.26 (0.95~2.36)	0.48 (0.32~1.03)	0.61 (0.38~1.05)	0.012

	**N4-acetylsulfamonomethoxine**					
	Range	< 4.23	4.23~4.33	4.34~4.88	≥4.89	
	Mode 1	1.00 (ref)	2.00 (0.05~78.25）	0.00 (0.00~∞)	1.00 （0.03~50.40）	0.251
	**Cefquinome sulfate**					
	Range	< 53.74	53.75~57.99	58.00~116.74	≥116.75	
VAs	Mode 1	1.00 (ref)	1.00(0.00~∞)	3.23E(0.00~∞)	2.61E(0.00~∞)	0.022
	**Ceftiofur**					
	Range	< 0.14	0.15~0.28	0.29~2.35	≥2.36	
	Mode 1	1.00 (ref)	1.00(0.00~∞)	3.23E(0.00~∞)	2.61E(0.00~∞)	0.022
	**Cyadox**					
	Range	< 218.44	218.45~232.49	232.50~377.49	≥377.50	
	Mode 1	1.00 (ref)	1.00(0.00~∞)	3.23E(0.00~∞)	2.61E(0.00~∞)	0.022
	**Levofloxacin**					
	Range	< 6.20	6.20~6.52	6.53~6.81	≥6.81	
	Mode 1	1.00 (ref)	3.23(0.00~∞)	8.08(0.00~∞)	1.62E(0.00~∞)	0.384
HAs	**Clarithromycin**					
	Range	< 0.91	0.91~0.93	0.94~0.95	≥0.96	
	Mode 1	1.00 (ref)	0.50(0.01~19.56)	2.00(0.05~78.25)	0.00(0.00~∞)	0.384

PVAs: preferred as veterinary antibiotics. VAs: veterinary antibiotics. HAs: human antibiotics.

Model 1: Elements were included in the conditional logistic regression models separately (single-antibiotic) and adjusted for age and gender.
