# Supplementary material for: The Abnormal Accumulation of Lipopolysaccharide Secreted by Enriched Gram-Negative Bacteria Increases the Risk of Rotavirus Colonization in Young Adults
Source: Microorganisms. 2023 Sep 11;11(9):2280. doi: 10.3390/microorganisms11092280 (PMC10535061; doi:10.3390/microorganisms11092280)
Supplement: Supplementary file 1 [file microorganisms-11-02280-s001.zip › Table S3.pdf]

Table S3: Adjusted ORs (95% CI) for RV colonization according to quartiles of the relative abundance of bacterial taxon and LPS level in fecal samples

		Quartile 1	Quartile 2	Quartile 3	Quartile 4	*p* trend
Enterococcus	Range (relative %)	< 2.97	2.97~3.89	3.90~11.94	≥11.95	0.0022
	Model 1	1.00 (ref)	2.45 (1.34-4.15)	1.38 (1.26-3.42)	2.78 (1.61-5.29)	
Enterococcaceae	Range (relative %)	< 6.47	6.48~7.09	7.10~7.32	≥7.33	0.499
	Model 1	1.00 (ref)	1.67 (1.00-3.21)	2.73 (1.27-4.01)	2.89 (1.62-5.34)	
Enterobacteriales	Range (relative %)	<11.54	11.55~12.14	12.15~13.49	≥13.50	0.028
	Model 1	1.00 (ref)	0.81 (0.56-1.24)	0.85 (0.51-1.32)	0.55 (0.35-0.94)	
Gammaproteobacteria	Range (relative %)	<1.04	1.05~1.29	1.30~3.14	≥3.15	<0.0001
	Model 1	1.00 (ref)	1.56 (0.95-2.79)	2.51 (1.42-4.58)	3.88 (2.47-6.29)	
LPS	Range (EU/mL)	<0.56	0.56-1.21	1.22-2.41	≥2.42	<0.0001
	Model 1	1.00 (ref)	1.25 (0.62-2.15)	1.41 (0.87-2.37)	2.69 (1.52-4.59)	

Model 1: Elements were included in the conditional logistic regression models separately (single- the relative abundance of bacterial taxon/LPS level) and adjusted for age and gender.
